# Editorial: Genetic, epigenetic and molecular landscaping of puberty

**DOI:** 10.3389/fendo.2023.1178888

**Published:** 2023-03-16

**Authors:** Sasha R. Howard, Pavlos Fanis, Nicolas C. Nicolaides, Anna Grandone

**Affiliations:** ^1^ William Harvey Research Institute, Barts and The London School of Medicine and Dentistry, Queen Mary University of London, London, United Kingdom; ^2^ Department of Paediatric Endocrinology, Barts Health NHS Trust, London, United Kingdom; ^3^ Department of Molecular Genetics, Function & Therapy, The Cyprus Institute of Neurology and Genetics, Nicosia, Cyprus; ^4^ First Department of Pediatrics, National and Kapodistrian University of Athens, Athens, Greece; ^5^ Department of Women, Children and General and Specialist Surgery, University of Campania Luigi Vanvitelli, Caserta, Italy

**Keywords:** puberty, central precocious puberty (CPP), delayed puberty onset, congenital hypogonadotropic hypogonadism, Klinefelter syndrome, epigenetics

The genomic landscape of puberty is a rapidly progressing area of pediatric endocrinology in which there have been major developments in our understanding over recent years. Given the importance of genetic, epigenetic, and molecular mechanisms in the regulation of puberty, this Research Topic aimed to collect new studies in this subject, from diverse fields including central precocious puberty, delayed puberty, hypogonadotropic hypogonadism, puberty in Klinefelter syndrome and Silver Russell syndrome, and environment-gene interactions in puberty

The article “*Pubertal timing in children with Silver Russell syndrome (SRS) compared to those born small for gestational age (SGA)*” presented an observational study of the pubertal and auxological features of patients with SRS as compared to patients with SGA alone. The authors, Patti et al., concluded that timing of puberty is affected in patients with SRS regardless of postnatal weight increase, and that puberty is earlier in patients with maternal uniparental disomy of chromosome 7 as compared to loss of methylation of chromosome 11p15.

In “*Dealing with Brain MRI Findings in Pediatric Patients with Endocrinological Conditions: Less Is More?*” the authors, Baldo et al., tackled a difficult clinical scenario of neurological imaging in children with pubertal disorders. Of particular challenge is the finding of incidental abnormalities on MRI brain scans in patients with central precocious puberty, such as arachnoid or pineal cysts, and the authors describe monitoring and follow up for these and other MRI findings in this group of patients.

The review article “*MKRN3 role in regulating pubertal onset: the state of art of functional studies*” summarizes and discusses some of the recent approaches developed to predict makorin ring finger protein 3 (MKRN3) functions and its involvement in pubertal development (Palumbo et al.). Loss-of-function mutations in this gene, in fact, represent the most commonly known genetic cause of central precocious puberty (CPP) but its role in pubertal onset control is not completely known.

In the work “*Navigating Disrupted Puberty: Development and Evaluation of a Mobile-Health Transition Passport for Klinefelter Syndrome*” the authors face the difficult problem of transition to adult care of patients with Klinefelter syndrome (Dwyer et al.). In particular they developed and tested a digital transition passport that was found to be usable, understandable, and had high ratings for actionability.

Faienza et al. provided a comprehensive review entitled “*Genetic, epigenetic and environmental influencing factors on the regulation of precocious and delayed puberty*” (5). In this article, the authors present the complex interaction of genes with environmental factors regulating pubertal timing. Indeed, defects in genes encoding kisspeptin receptor (*KISS1R*), Makorin Ring Finger Protein 3 (*MKRN3*) and Delta-like 1 homolog (*DLK1*), have been implicated in central precocious puberty, whereas mutations in a growing number of genes, such as *FGFR1*, *GNRHR*, *HS6ST1* and many others, contribute to delayed puberty. In addition to genetic defects, the authors discuss the role of epigenetics (DNA methylation and microRNAs, miRNAs) in the onset of puberty. Finally, Faienza et al. highlight the emerging involvement of endocrine disrupting chemicals, as environmental factors, in regulating pubertal initiation.

In the research article “*Correlation Analysis of Genotypes and Phenotypes in Chinese Male Pediatric Patients with Congenital Hypogonadotropic Hypogonadism*” Wang et al. studied the medical records of 125 Chinese male patients aged 0–18 years with congenital hypogonadotropic hypogonadism (CHH). The authors collected the clinical characteristics, the hormonal measurements, and the genetic defects of the participants through whole-exome sequencing, and performed a correlation analysis of genotypes and phenotypes. Importantly, they also found 15 new CHH-related genes, compared to previously published studies. Finally, Wang et al. concluded that cryptorchidism, micropenis and the genetic defects are *sine qua non* factors for early and accurate diagnosis of CHH in children and adolescents, and further discussed the long-term follow up of these patients.

In the article “*Integrated analysis of proteomics and metabolomics in girls with central precocious puberty*”, the results of proteomics and metabolomics in serum samples from girls with CPP are presented in an attempt to identify potential biomarkers (Li et al.). Bioinformatic analyses led to the identification of 134 differentially expressed proteins in girls with CPP with 71 upregulated and 63 downregulated proteins and the identification of 103 differentially expressed metabolites, including 42 upregulated and 61 downregulated metabolites. By performing network analysis of integrated proteomics and metabolomics, the authors revealed lipid metabolic pathways that may be involved in pubertal development in girls.

The review article “*Genetic architecture of self-limited delayed puberty and congenital hypogonadotropic hypogonadism*” examines the distinction between self-limited delayed puberty and congenital hypogonadotropic hypogonadism (Vezzoli et al.). The authors provide an updated overview of the genetics behind these two conditions and discuss the advantages and disadvantages of genetic analysis, particularly since the introduction of next generation sequencing, to effectively distinguish between these two conditions.

Together, these articles demonstrate the fantastic complexity of the genetic and epigenetic control of puberty, and the importance of the interaction of molecular, genomic and clinical aspects in the etiology of pubertal disease. Understanding of the genetic and epigenetic mechanisms driving pubertal disorders can benefit diagnosis and therapeutic management for patients with these conditions, and also open up new avenues for basic science exploration to promote identification of important molecular mechanisms and associated pathways. Digital technologies, whether radiological tools or the use of mobile phones for health passports can also revolutionize patient care. But amongst all this new discovery, the basics of clinical care, such as precise phenotyping of patients to aid diagnosis and attendance to the patients’ holistic and psychosocial needs, remain vital to support best outcomes for patients with disorders of puberty.

## Author contributions

All authors listed have made a substantial, direct, and intellectual contribution to the work and approved it for publication.

